# Machine learning outperforms clinical experts in classification of hip fractures

**DOI:** 10.1038/s41598-022-06018-9

**Published:** 2022-02-08

**Authors:** E. A. Murphy, B. Ehrhardt, C. L. Gregson, O. A. von Arx, A. Hartley, M. R. Whitehouse, M. S. Thomas, G. Stenhouse, T. J. S. Chesser, C. J. Budd, H. S. Gill

**Affiliations:** 1 Present Address: Institute for Mathematical Innovation, University of Bath, Bath, UK; 2 Musculoskeletal Research Unit, Bristol Medical School, University of Bristol, Bristol, UK; 3 MRC Integrative Epidemiology Unit, Bristol Medical School, University of Bristol, Bristol, UK; 4 Royal United Hospital NHS Foundation Trust, Bath, UK; 5 Department of Trauma and Orthopaedics, North Bristol NHS Trust, Bristol, UK; 6 Department of Mathematical Sciences, University of Bath, Bath, UK; 7 Department of Mechanical Engineering, University of Bath, Bath, UK; 8 Centre for Therapeutic Innovation, University of Bath, Bath, UK

**Keywords:** Trauma, Computational science

## Abstract

Hip fractures are a major cause of morbidity and mortality in the elderly, and incur high health and social care costs. Given projected population ageing, the number of incident hip fractures is predicted to increase globally. As fracture classification strongly determines the chosen surgical treatment, differences in fracture classification influence patient outcomes and treatment costs. We aimed to create a machine learning method for identifying and classifying hip fractures, and to compare its performance to experienced human observers. We used 3659 hip radiographs, classified by at least two expert clinicians. The machine learning method was able to classify hip fractures with 19% greater accuracy than humans, achieving overall accuracy of 92%.

## Introduction

Hip fractures are a major cause of morbidity and mortality for the elderly, and incur high direct health costs^1^. In 2019, 67,671 hip fractures were reported to the UK National Hip Fracture Database^2^; given the projections for population ageing over the coming decades, the number of hip fractures is predicted to increase globally, particularly in Asia^3–5^. Currently, across the world, an estimated 1.6 million hip fractures occur annually with substantial economic burden, approximately $6 billion per year in the US^6^ and about £2 billion in the UK^7^. Patients who sustain a hip fracture have a reported 30-day mortality of 6.9% in the UK in 2019^8^, with 30% of patients dying over the course of the first year, i.e. twice the age-specific mortality rate of the general population^9,10^. Thus, the development of strategies to improve hip fracture management and hence their impact on mortality and healthcare provision costs is a high priority^10,11^.

**Figure 1:**
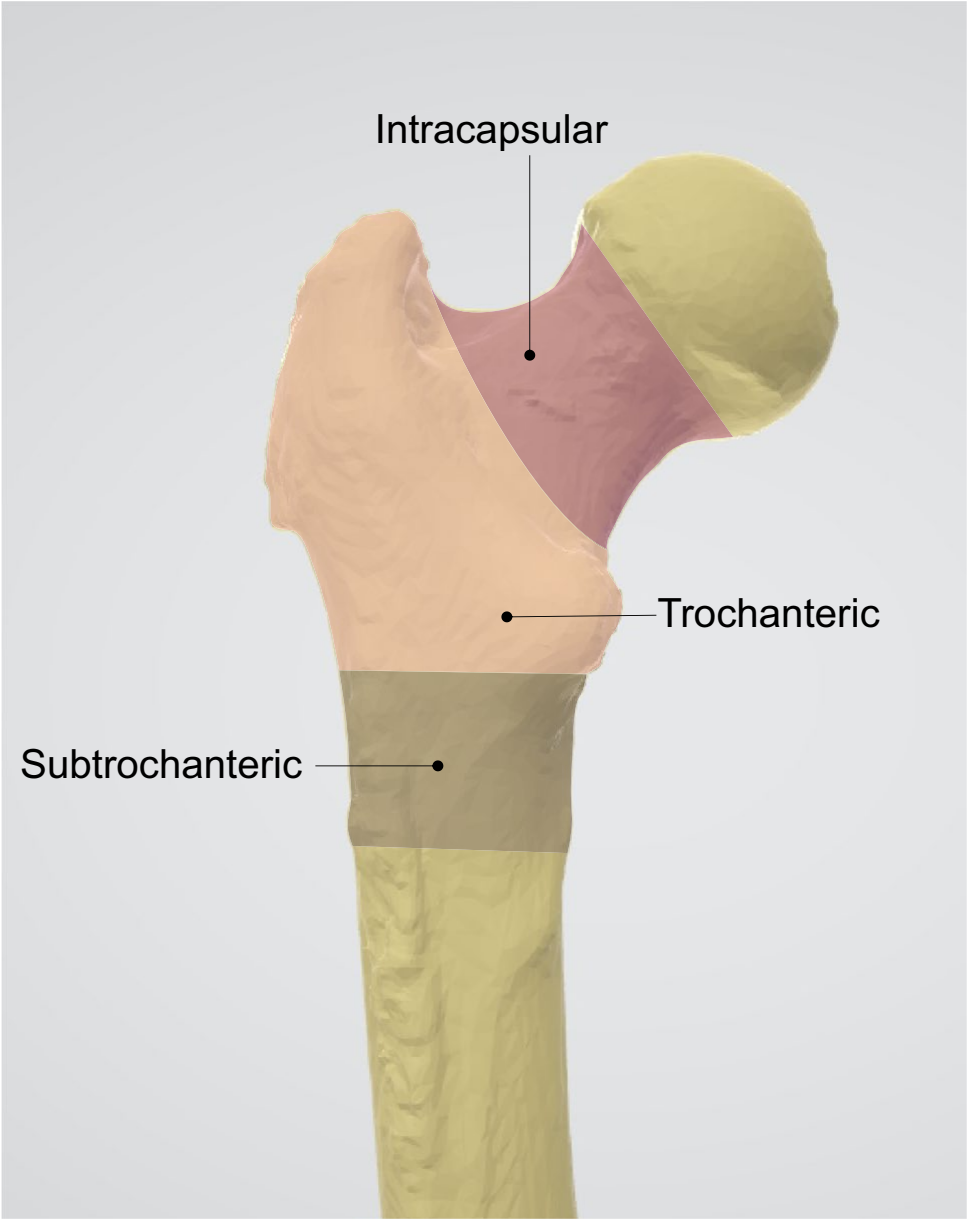
Hip fracture types.

When patients suffer a hip fracture, treatment aims are to restore function and relieve pain whilst minimising risk of morbidity and mortality, hence 98% of hip fractures are managed operatively in the UK^8^. Surgical treatment of hip fractures is strongly influenced by the fracture type^12,13^. Hip fractures can be classified using the AO system^14^, or by describing the fracture location and displacement with a modification of this system, as used by the UK Falls and Fragility Fracture Audit Programme (FFFAP)^15^ in their National Hip Fracture Database (NHFD) clinical audit^16^. Figure 1 illustrates the three main classes of hip fractures: intracapsular, trochanteric (extracapsular), and subtrochanteric (extracapsular). The AO system further defines subclasses: Grade A1/A2 and Grade A3 for trochanteric fractures accordingly to trochanteric area involvement and the presence of displacement for intracapsular fractures. There are recognised limitations with the current methods used for the classification of fractures^17^. Interobserver agreement is slight to fair whether using the original or new AO classification systems^18^ and fair to substantial for the NHFD classification system^19^.

Fracture classification, according to these methods, aids surgeons in selecting the right surgical interventions to treat the fracture to restore mobility. The choice of operation and implant has a strong influence on treatment costs; for example sliding hip screws and intramedullary nails are two of the treatment options for trochanteric fractures but the cost of intramedullary devices is 3 to 4.5 times higher than for sliding hip screws^13^. Furthermore, the choice of intervention for a given fracture type predicts the risk of death following surgery^20^. Hence governance bodies such as the National Institute for Health and Care Excellence (NICE) place great emphasis on the choice of operation and implant that should be offered for different hip fracture types, reflecting both the evidence-base and the potential cost of some implant types^13^, such that NICE compliant surgery is one of the six key performance indicators for the provision of hip fracture care in the UK^7^.

Despite fracture classification so strongly determining surgical treatment and hence patient outcomes, there is currently no standardised process as to who determines this classification in the UK (e.g. orthopaedic surgeon or radiologist specialising in musculoskeletal disorders).

A critical issue affecting the general use of diagnostic imaging is the mismatch between demand and resource. The total number of imaging and radiological examinations has steadily increased, for example the number of radiographs performed annually has increased by 25% from 1996 to 2014^21,22^. The increasing demand on radiology departments often means that they cannot report all acquired radiographs in a timely manner. In the UK it is estimated that more than 300,000 radiographs remain unreported for over 30 days^23^. Annarumma et al. demonstrated how a machine learning approach can support hospitals to dramatically cut time needed to process abnormal chest radiographs^23^. For hip fracture management, the ability to accurately and reliably classify the fracture swiftly is paramount as surgery should occur within 48 h of admission^13,24–26^, because delays in surgery increase the risk of adverse patients outcomes such as mortality^27^.

Machine learning methods offer a new and powerful approach by which to automate diagnostics and outcome prediction across a diverse set of medical disciplines and pathologies: from oncology^28–31^ and radiology^32^, to diabetes treatment^33^ and rheumatology^34,35^. Beside advances in computing power, one key to the success of machine learning has been the development of convolutional neural networks (CNNs)^36–38^. For example state- of-the-art performance in estimating bone age from hand radiographs^39^ and detecting knee joints^34^. Krogue et al.^40^ were able to demonstrate that machine learning could classify hip fractures based on radiographs of 972 patients. Following these successes, we aimed to create a machine learning method for identifying and classifying hip fractures on plain radiographs acquired as part of routine clinical care to determine if this method can outperform trained clinical observers in identifying and classifying hip fractures.

## Results

The results are presented in terms of the variables introduced in the Methods section.

### CNN1: automatically locating the hip joint

**Figure 2:**
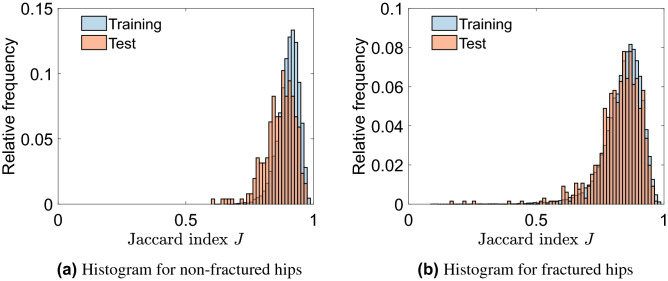
Performance assessment of CNN1 based on the Jaccard index *J*, which measures the agreement between two images. *J* = 0 means no agreement and *J* = 1 means total agreement; *J* > 0.5 is considered good agreement.

CNN1 was able to correctly locate and extract hip joints in the vast majority of cases. This was true for both fractured and non-fractured hips; with the performance on radiographs of non-fractured hips being slightly better than those of fractured hips (Fig. 2). The Jaccard index *J* had higher values for the training sets than for the corresponding test sets. To assess the overall performance of the machine learning method, the Jaccard Index for the test data is most relevant. For the test data of Dataset 1, the mean value of *J* was 0.87 (SD 0.06), all samples scored values of *J* > 0.5 and 98% of the hip joints scored *J* > 0.7 (indicating better than good agreement). For the test data of Dataset 2, the mean value of *J* was 0.83 (SD 0.09), more than 99% of the test set scored a value of *J* > 0.5, with 93% exceeding a value of *J* > 0.7. This implies that CNN1 was able to extract the region around the hip joints with very close alignment to the ground truth region of interest.

### Expert agreement in fracture classification

**Figure 3:**
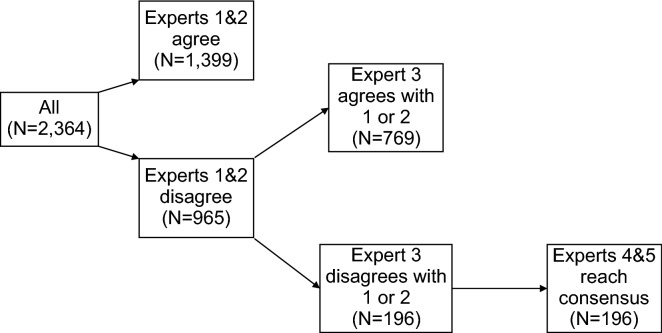
Expert fracture classification process and agreement for Dataset 2.

Agreement between expert clinicians was used to assign the ground truth label to each radiograph. Experts only agreed on the category (i.e. subclass) in 1399 cases (59.2%), leading to a Cohen’s Kappa *κ* = 0.49 (95% CI: 0.47 to 0.52) (Fig. 3). Comparing the overall class (instead of the subclasses) assigned to a radiograph, the first and second experts agreed in 1,663 cases (70.4%) (*κ* = 0.55, [95% CI: 0.52 to 0.58]).

### Hospital diagnosis compared to expert classification

Within Dataset 2, 2,181 radiographs had fracture type recorded, which was termed the hospital diagnosis. When compared to the expert classification (Table S1 in the Supplementary Material), which was considered as the ground truth, the hospital diagnosis had an overall accuracy of 77.5% (*κ* = 0.63, [95% CI: 0.61 to 0.66]).

### CNN2: classification of hip fractures test set

**Figure 4:**
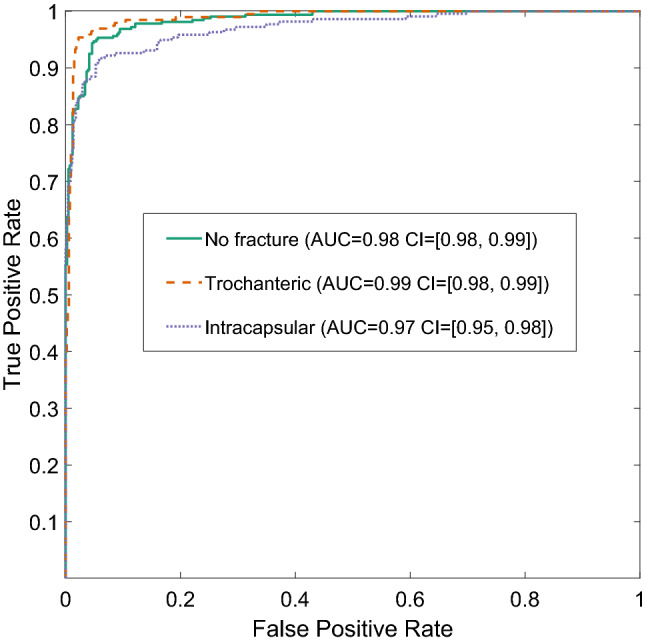
Receiver Operating Characteristic (ROC) curves illustrating trade-offs between true-positive and false-positive rate for the three classes of hip fracture, as predicted by CNN2 using AUC = area under the curve, given with the 95% confidence interval (CI).

CNN2 predicted the correct fracture type in 92% (which represents the overall accuracy) of the test set (*κ* = 0.87 [95%CI: 0.84 to 0.90]: NB Cohen's kappa, *κ,* varies from *κ* = 1 for complete agreement to *κ* = 0 if the agreement is no better than expected by chance). This represents an 18.7 (= 100*[92–77.5]/77.5) percentage points increased accuracy over the original hospital diagnosis accuracy. The precision varied between 0.87 for intracapsular fractures to 0.96 for trochanteric fractures (Table 1). Similarly, recall varied between 0.87 for trochanteric fractures and 0.95 for no fractures (Table 1). The confidence intervals between the best predicted and worst predicted class did not overlap, indicating that there were significant differences between the performance of the best and the worst classes in precision and recall. Combining precision and recall led to per-class F1 scores of 0.94, 0.91 and 0.89 for no fracture, trochanteric fracture and intracapsular fracture, respectively (Table 1). Figure 4 displays the Receiver Operating Characteristic (ROC) curves for all three classes with the corresponding area under the curve (AUCs) and their 95% confidence intervals. We observed AUCs of 0.98 (95% CI: 0.98 to 0.99) for “No fracture”, 0.99 (95% CI: 0.98 to 0.99) for “Trochanteric” and 0.97 (95% CI: 0.95 to 0.98) for “Intracapsular”.

**Table 1:** CNN2 performance assessment.

	Actual			Total
	No fracture	Trochanteric	Intracapsular	
**Predicted**				
No fracture	304	12	13	329
Trochanteric	1	169	6	176
Intracapsular	15	14	198	227
Total	320	195	217	
Precision	0.92	0.96	0.87	
95% CI	0.89 to 0.95	0.92 to 0.98	0.82 to 0.91	
Recall	0.95	0.87	0.91	
95% CI	0.92 to 0.97	0.81 to 0.91	0.87 to 0.95	
F1	0.94	0.91	0.89	

Precision = (number correctly predicted as class A)/(number predicted as class A). Recall = (number correctly predicted as class A)/(number actually of class A). F1 varies from 1 = perfect classifier for class A, to 0 = no image was correctly identified as class A.

**Figure 5:**
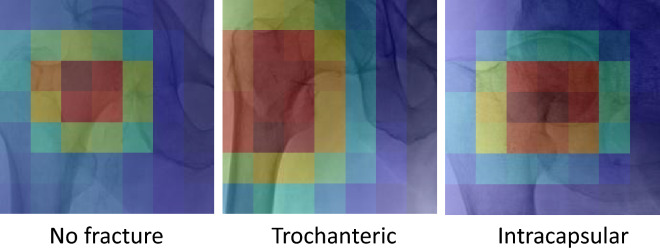
Activation maps for representative examples for No fracture, Trochanteric and Intracapsular classes. Dark red implies regions of high contribution and dark blue regions of low contribution. A custom python code based on the code provided by Selvaraju et al.^41^ downloaded from github (https://github.com/ramprs/grad-cam) was used to generate the activation maps.

Activation maps (Fig. 5) for representative examples of each of No fracture, Trochanteric and Intracapsular provided an insight into the parts of the x-ray image contributing to the classification. For the No fracture the centre of the femoral neck region was highlighted. The region distal and lateral to the neck was highlighted for the Trochanteric. Finally, for the Intracapsular the region distal and medial to head was highlighted.

## Discussion

Hip fracture remains a common and devastating injury that places substantial pressures on healthcare systems around the world. The aim of the current study was to create a machine learning algorithm to identify and classify hip fractures. The work successfully produced two convolutional neural networks, one to automatically localise the hip joints within an AP pelvic radiograph (CNN1) and one to identify and classify the type of fracture within an AP radiograph of a hip joint (CNN2).

The hip joint localisation algorithm (CNN1) was highly accurate in locating hip joints, whether the joint was fractured or not. One hundred percent of the test set for the non-fractured dataset (Dataset 1), and 99% of the test set of the fractured dataset (Dataset 2) showed a Jaccard index *J* > 0.5 considered a good agreement, and 98% and 93% even exceeded *J* = 0.7 (very good agreement), respectively.

The classification algorithm showed an impressive, and potentially significant, performance with an AUC > 0.97 for all three classes. It is important to note that the radiographs used were acquired as part of routine clinical care with an NHS hospital setting, with variable quality due to the acute nature of the injury. The overall accuracy was 92%; a significant improvement (test for the difference of independent proportions, p-value < 0.0001) compared to human observers who had an accuracy of 77.5% in the original hospital diagnosis. While there were significant differences in precision and recall between the three classes, each class was very good in either precision or recall, leading to high F1 scores. Furthermore, there was no significant correlation between the number of experts needed to agree on the actual class and whether the radiograph was correctly classified by the machine learning algorithm (Chi-Square test: *p* = 0.65). This indicates that human observers and the machine learning algorithm did not find the same fractures challenging to classify. Having said that, a radiograph was classified by an additional expert if the experts disagreed on the subclass while the machine learning algorithm only classified into classes. The machine learning algorithm correctly identified significantly more of the non-fractured hip joints than for any other fracture type suggesting that this is the easiest class for the machine learning algorithm.

Machine learning has been used previously for detecting hip fractures, Adams et al.^42^ used a CNN trained on 640 images with 160 images for validation for detecting hip fractures, and were able to show accuracy of 94.4%. Chen et al.^43^ also used a CNN for detecting hip fractures, the CNN was trained on 3605 pelvic x-rays and evaluated on 100 pelvic x-rays, they reported an accuracy of 91%. A different approach was taken by Badgeley et al.^44^, who used images as well as patient and hospital data to “predict” hip fracture, and reported an accuracy of 85% in detecting fracture. In terms of using CNNs for classification, Krogue et al.^40^ used radiographs from 972 patients, they reported a classification accuracy of 90.8% but only had a comparison of 100 radiographs assessed by two residents. Yamada et al.^45^ reported 98% accuracy in classifying femoral neck fractures, trochanteric fractures, and non-fracture with an accuracy using a combination of AP and lateral x-rays, their CNN was trained on 1,553 AP hip radiographs and 1,070 lateral hip radiographs and validated on 150 AP and lateral hip radiographs. Yamada et al. concluded that using both AP and lateral x-rays improved accuracy, however in many clinical centres in the UK lateral x-rays are not available. The current study differed from previous studies in that all available clinical x-rays were used, regardless of quality whilst other studies excluded poor quality x-rays. This is an important consideration in working towards a clinically useful tool, we believe that excluding low quality x-rays artificially inflates accuracy. Previous studies did not report fully how the training sets were classified and level of clinical agreement, most studies did report how the test sets were evaluated. The current study went to considerable lengths to have consensus classification for all x-rays used in the study, for training, validation and test. We report that the first two clinical reviewers only agreed on sub-class for 60% of the cases, requiring further rounds of clinical classification to reach consensus. The current study also used considerably larger validation and test data sets, which consisted of 732 x-rays for each.

Due to the negative consequences of a hip fracture misclassification, we further investigated a more conservative approach where we only classified an image if the algorithm’s confidence score was greater than a threshold. While this led to some radiographs not being classified, it also increased the accuracy on the classified images. In practice, for the remaining non-classified images an expert’s opinion would be needed. There is a trade-off between overall accuracy and coverage (% of classified images). For example, if an accuracy of 95% was required (we currently achieved 92%), 87% of the data set would be covered, while the remaining 13% would not be classified by the algorithm. Furthermore, we could set different demands for different fracture types. The treatment differs between the classes of hip fractures in how invasive they are and in cost for the NHS. One could demand more certainty for some classes than others by setting different thresholds for the scores and leaving uncertain radiographs to be analysed by an expert.

Radiographs of patients with a hip fracture may not be of high quality. Patients are in pain following a hip fracture and approximately a third of the population affected have cognitive impairment^46^ making it challenging for them to follow instructions from radiographers in terms of positioning for radiograph acquisition. Automated settings applied by digital radiography systems may also affect the ability to interpret radiographs^47^. This can lead to low quality images that are difficult for clinicians to interpret. Clinicians may also follow different criteria for fracture classification according to their training and prior experience of interpreting radiographs and treating hip fracture. This may lead to variation in their interpretation of the same image. This variation in classification and the problems it creates in treating hip fractures are well recognised^18,19^. A pre-established automated classification system may improve accuracy of diagnosis of the basis of plain radiographs, which are routine clinical practice in this population worldwide. The activation mapping provided some insight into the regions of the x-ray images contributing to each type of classification. For the trochanteric and intercapsular examples, as expected regions that contained the fracture contributed the most. Interestingly, for the No Fracture case, the central part of the femoral neck contributed the most.

Introduction of a system capable of accurate and reproducible classification of radiographs of patients with a hip fracture would allow the delivery of accurately targeted surgical interventions. Importantly it would reduce the chance of changes to the surgical plan, which can delay the delivery of treatment to the affected and other patients, and reduce unwarranted delay to surgery to seek information from further imaging which may be associated with increased risk of morbidity and mortality for patients^27,48^. Such a system would also aid the standardisation of comparative studies, interpretation of large healthcare datasets, and the delivery and interpretation of clinical studies where the population, exposures and covariates may depend upon the accurate classification of hip fractures^19^.

A limitation of our method was that we excluded subtrochanteric fractures due to the lack of available data.

## Conclusion

In this work, we have demonstrated that a trained neural network can classify hip fractures with 19% increased accuracy compared to human observers with experience of hip fracture classification in a clinical setting. In the work presented here, we used as ground truth the classification of 3,659 hip radiographs by at least two (and up to five) experts to achieve consensus. Thus, this analysis is a prototype only and a more extensive study is needed before this approach can be fully transformed to a clinical application. We envisage that this approach could be used clinically and aid in the diagnosis and in the treatment of patients who sustain hip fractures.

## Methods

All methods were carried out in accordance with relevant guidelines and regulations.

### Data sources

We used two different populations to source antero-posterior (AP) pelvis radiographs; note pelvis radiographs visualise both hip joints. The first (Dataset 1) was a population in which no hip fractures had occurred and consisted of 429 anonymized radiographs collected as part of an ethically approved (REC: 05/Q2001/78), with informed consent obtained from all participants, multi-centred observational study of bone mass^49^. The population comprised adults with mean age (± SD) 61.9 ± 12.0 years, 64% were female, none had a hip fracture.

Dataset 2 consisted of 2,364 anonymized AP pelvic radiographs from patients admitted acutely to a National Health Service (NHS) hospital who were diagnosed with a hip fracture. This population was identified from local National Hip Fracture Database (NHFD) audit records which included all hip fractures admitted between 2008 and 2016 (mean age 80 ± 10 years, 70% female). Ethical approval (Ref: 2017 0299 05, Royal United Hospital R&D Ethics Committee) was obtained for anonymous re-use of radiographs, as fully anonymised existing data were used informed consent was not required. Using these 8 years of audit record, which included fracture type, radiographs were selected by stratified random sampling to oversample less common fracture types. It is important to note that radiograph quality was not used as a selection criterion, the dataset was representative of the range of image quality in clinical radiographs taken in an acute setting. The recorded fracture type, termed the hospital diagnosis, was present for 2,181 radiographs. All radiographs were examined and hip fractures re-classified by at least two musculoskeletal experts using the National Hip Fracture Database classification^8^ (Table 2); in this study the final classification by the musculoskeletal experts was considered the ground truth. The accuracy of the hospital diagnosis was established by comparing with this ground truth. The non-fractured contralateral hip images were also used, provided no implant was in situ. Hence this dataset contained 1,603 non-fractured hip images, with 1,089 intracapsular, 993 trochanteric, and 114 subtrochanteric fractures visible. A further 168 radiographs could not be classified.

**Table 2:** Ground-truth classification according to musculoskeletal experts for Dataset 2.

Class	NHFD subclass	Subtotal	Total
Intracapsular	Displaced	864	1089
	Undisplaced	207	
	Unable to determine subclass	18	
Trochanteric	Grade A1/A2	818	993
	Grade A3	151	
	Unable to determine subclass	24	
Subtrochanteric			114
Unfractured			1603
Not classifiable			168

### Image processing

The radiographs were obtained in DICOM format, the average size of the AP radiographs was (HxW) 2,186 (SD: 223) × 2460 (SD: 255) pixels. Typically, CNNs process images of a much smaller size, e.g. 200 by 300 pixels. To retain as much useful information as possible and discard the areas of the image that do not assist in diagnosis/classification, we introduced a first stage where we trained a CNN (CNN1) to automatically extract two regions of interest (ROI), each containing a hip joint. This reduced the radiograph size to two ROIs of about a quarter of the original size of the radiograph, i.e., approximately 1200 × 1000 pixels. We then down sampled the ROIs using bicubic interpolation and antialiasing to provide images of 256 × 256 pixels (MATLAB R2016b, The MathWorks Inc, Natick, MA, USA). Each down-sampled ROI was then passed to a second CNN (CNN2) which was trained to determine if a fracture was present and if so, classify the type of hip fracture.

### Training, testing and validation data

When creating both CNNs the available data sets were split at random into three sub-sets: training (60%), validation (20%) and test sets (20%)^50^. This allowed us to assess the performance of our analysis methods on a test set which was independent of the data upon which it was trained and validated, ensuring rigor in machine learning^50,51^. To artificially increase the size of the training sets by a factor of 16, standard operations (flipping, inverting and random shifts) were applied to generate more training images. This is an established procedure for increasing size of training data sets. In addition, to address the imbalance in fracture types within Dataset 2 training set, less frequent fracture types (intracapsular and trochanteric) were oversampled so that the numbers of each fracture type in the training set were similar. Note neither of these operations were applied to the validation and test sets. For Dataset 1 the final number of training/validation/test images were 6128/128/127 respectively; whilst for Dataset 2 these were 47,698/732/732 respectively.

### CNN1: automatically locating the hip joint

Radiographs of fractured hips (taken in an acute setting) were found to be much more variable in terms of quality and patient positioning (Fig. S1). For efficient CNN training, we therefore, first trained CNN1 on non-fractured hips (Dataset 1, taken in a scheduled clinic setting) where the radiographs were more homogeneous and then fine-tuned the resultant CNN using radiographs of fractured hips (Dataset 2).

Radiograph images were labelled manually (by EM); then the ROI containing each hip joint was identified (MATLAB Training Image Labeller Application, the MathWorks Inc.). To have consistent ROIs, natural features of the hip joint were selected as boundary markers (Supplementary Material and Fig. S2), chosen based on expert orthopaedic surgical opinion, ensuring that the ROIs provided sufficient coverage of the hip joint to enable classification to be performed. The ROIs were used to create a mask for each image.

The success of Antony et al.^34^ in locating knee joints on radiographs inspired us to use a fully convolutional network (FCN) to detect the ROIs. FCNs were developed to allow for pixel-wise classification (i.e., semantic segmentation^52^) where each pixels was classified as either in or outside of the ROIs. First, the network was trained on Dataset 1. Second, this trained network was then retrained on the Dataset 2. As FCN ROIs were rough-edged, they were converted to a rectangular shape by post-processing contour detection (MATLAB).

To assess the performance of CNN1, we compared the ground-truth labels with the predicted labels for each radiograph in the test set using the Jaccard Index, *J*, which measures the agreement between two images^53^. The Jaccard index varies between 0 (no agreement) and 1 (total agreement). By convention, the predicted ROI is considered to be “correct” for a value of *J* > 0.5^34,54^.

### CNN2: classification of hip fractures

#### Labelling, data preparation and augmentation

To determine the ground-truth labels for the radiographs, all Dataset 2 radiographs were read and classified independently by two musculoskeletal experts (consultant orthopaedic surgeon and/or consultant musculoskeletal radiologist) blinded to patient details. Each expert routinely assesses hip fractures as part of their day-to-day work. The experts were able to choose one of eight possible labels listed in the first two columns of Table 2. If the two experts did not agree on classification for a given radiograph, independent classification by a third expert was performed. If this agreed with one of the two original classifications, this classification was used else the radiographs were jointly read and classification agreed by two further experts with the most experience (MW, TC).

The frequencies of the NHFD subclasses of intracapsular and trochanteric fracture types varied greatly, with some subclasses seen on as few as 18 radiographs. As CNNs have difficulty learning from unbalanced and small classes, subtrochanteric fractures and non-classified fractures were excluded. Thus, we limited CNN training to the classification of three classes: no fracture, trochanteric and intracapsular fractures. Radiographs of hips with an implant in situ were excluded from the dataset, leaving 1082 intracapsular, 974 trochanteric and 1603 non-fractured (n = 3659 total). The training set was augmented and artificially increased in size by rotating the images through an angle between -10° and 10°, chosen at random. Furthermore, half of the images were grayscale-inverted (chosen at random) to exploit the fact that some radiographs use an inverse intensity scheme: light illustrating bone and dark tissue. The network randomly mirrored images in the training set, so this was not repeated. The minority classes were sampled more frequently to address the class imbalances, resulting in a training set size of 47,698, evenly spread across fracture types. No augmentation was applied to the validation and test sets which were of size 732 (320 non-fractured, 195 trochanteric and 217 intracapsular).

#### Training: transfer learning with GoogLeNet

The number of radiographic images available in the classification training set was small by traditional machine learning classification tasks, which risks reduced accuracy. A standard solution is transfer learning: utilizing a network that has already been trained to a high degree of accuracy on a similar task using a much larger dataset. This lessens overfitting, improves accuracy and greatly reduces the time needed to train a network^50,51^.

For the transfer learning for the classification task, we exploited the success of GoogLeNet, a very successful model architecture developed by Google^55^. Instead of training the GoogLeNet network architecture from scratch, the Berkeley Vision and Learning Centre (BVLC) pretrained model^56^ was used as the starting point for finetuning CNN2 on Dataset 2. The details of how this model was trained are given in github.com/BVLC/caffe/tree/master/models/bvlc_googlenet.

The input for the final trained CNN2 was an AP radiograph of a hip joint, the output was a score for each fracture type considered (no fracture, trochanteric and intracapsular fractures) indicating the relative certainty that the hip belonged to the corresponding class. We assigned hips to the class with the highest score.

#### Performance assessment

The overall performance of the fracture classification was assessed using accuracy (fraction of predictions the model classified correctly) and agreement (Cohen’s kappa, κ)^57^. Cohen’s kappa coefficient, a statistic which measures the agreement between two labelling approaches, is more robust than accuracy since it contrasts accuracy with results accomplished when assigning labels at random. Cohen’s kappa coefficient varies from κ = 1 for complete agreement to κ = 0 if the agreement is no better than expected by chance.

Fracture-specific performance was also assessed by comparing the actual versus predicted labels for each class, which is commonly used in machine learning^50^. These were then summarized in precision (i.e. the number of all fractures correctly classified that were labelled with the same fracture type) and recall (i.e. the proportion of fractures of a given type that were correctly classified). Precision and recall for a fracture type A are defined as:

1 $$Precision = \frac{number\;correctly\;predicted\;as\;type\;A}{{number\;predicted\;as\;type\;A}}$$

2 $$Recall = \frac{number\;correctly\;predicted\;as\;type\;A}{{number\;actually\;of\;type\;A}}.$$

The 95% confidence intervals were calculated for precision and recall using the Clopper-Pearson method. The F1 score enabled combination of precision and recall into a single performance measure per class:

3 $$F1 = \frac{2}{{\left( {\frac{1}{precision} + \frac{1}{recall}} \right)}}.$$

F1 varies from 1: perfect classifier for type A; to 0: no image was correctly identified as type A.

Additionally, performance of the classification was assessed using Receiver Operating Characteristic (ROC) curves. As mentioned above, CNN2 assigns each image of a hip joint three scores between 0 and 1, one per class indicating the relative certainty that the hip belongs to the corresponding class. A threshold was then defined for the score needed to reach a decision. For instance, if an image is classified as class A only if the corresponding score exceeds 0.9, the images labelled A are more likely to be classified correctly, but there will be many incorrect “not A” labels. For each class and threshold, the number of correct and incorrect classifications were computed. Then ROC curves per class were plotted: the true positive rate, i.e. recall, versus the rate of false negatives. The threshold reflects the trade-off between recall and precision. Computing the area under the ROC curve (AUC) measures the overall performance of the classification algorithm, independent of the choice of threshold. An area of 1 represents a perfect classification, while an area of 0.5 is the same as a classification by chance. The 95% confidence intervals (CI) of the AUC were computed using 1000 bootstrap samples.

We used gradient-weighted class activation mapping (Grad-CAM^41^) to produce visual explanations for our model. Grad-CAM propagates the gradients of a particular classification (in our case, the predicted label) back to the final convolutional layer of the network to produce a heatmap illustrating the regions of the image that contributed most strongly to that classification. By definition, our visualisation heatmaps had the same resolution as the feature maps in the final convolutional layer, 7 × 7. A custom python code based on the code provided by Selvaraju et al.^41^ downloaded from github (https://github.com/ramprs/grad-cam) was used to generate the activation maps.

Fitting the CNNs was performed using Caffe (Berkeley Artificial Intelligence Research, University of California at Berkley, CA, USA)^58^, other computations were performed in MATLAB; except for the statistical tests which were performed using R^59^. The machine learning was performed on a Xeon workstation with a Titan X GPU (Nvidia Corporation, Santa Clara, CA, USA) running a Linux operating system.

## Supplementary Information

Supplementary Information.

## Data Availability

The datasets generated during and/or analysed during the current study are available in the University of Bath Research Data repository, 10.15125/BATH-01011.
