# Supplementary material for: Machine learning outperforms clinical experts in classification of hip fractures
Source: Sci Rep. 2022 Feb 8;12:2058. doi: 10.1038/s41598-022-06018-9 (PMC8825848; doi:10.1038/s41598-022-06018-9)
Supplement: Supplementary file 1 — Supplementary Information. [file 41598_2022_6018_MOESM1_ESM.docx]

# Supplementary Material

## Radiograph Quality

Figure S1: Contrasting the variability in radiograph quality of non-fractured hips (a, Dataset 1) with those of fractured hips (b, c, Dataset 2).

## Definition of ROIs for Labelling

ROIs were defined based on anatomical features to ensure consistency between images (Figure S2). The horizontal boundaries were the most lateral aspect of the greater trochanter and the most medial edge of Shenton’s line. The upper boundary marker was just proximal to the superior aspect of the acetabulum. The lower femur boundary was chosen such that the distance from the lower boundary to the most distal point of the greater trochanter (y) was approximately the same as the distance from the most distal to the most proximal points of the greater trochanter (x).

Figure S2: Boundaries of the ROIs for labelling

## Hospital Diagnosis Compared to Expert Ground Truth

|  |  | Hospital Diagnosis | | | |  |
| --- | --- | --- | --- | --- | --- | --- |
|  |  | Trochanteric | Intracapsular | Subtrochanteric | Other | Total |
| Ground Truth (expert classification) | Trochanteric | 697 | 43 | 167 | 5 | 912 |
|  | Intracapsular | 90 | 898 | 15 | 7 | 1010 |
|  | Subtrochanteric | 13 | 1 | 93 | 1 | 108 |
|  | Not Classifiable | 66 | 53 | 29 | 3 | 151 |
|  | Total | 866 | 995 | 304 | 16 | 2181 |

Table S1: Comparison between the original diagnosis in hospital (Hospital Diagnosis), available for 2,181 radiographs, and the ground truth (based on expert classification), accuracy: 77.5%.
